# Association of potential biomarkers with clinical outcomes in metastatic triple-negative breast cancer treated with pembrolizumab or chemotherapy

**DOI:** 10.1038/s41523-025-00814-y

**Published:** 2025-10-02

**Authors:** Javier Cortes, Oleg Lipatov, Seock-Ah Im, Anthony Goncalves, Keun Seok Lee, Peter Schmid, Kenji Tamura, Laura Testa, Shoichiro Ohtani, Nadia Harbeck, Sherene Loi, Roberto Salgado, Vassiliki Karantza, Jaime Mejia, Razvan Cristescu, Andrey Loboda, Michael Nebozhyn, Petar Jelinic, Lingkang Huang, Eric P. Winer

**Affiliations:** 1 International Breast Cancer Center (IBCC), Pangaea Oncology, Quironsalud Group, and IOB Madrid, Hospital Beata Maria Ana, and Universidad Europea de Madrid, Faculty of Biomedical and Health Sciences, Department of Medicine, Madrid, Spain; 2 Republican Clinical Oncology Dispensary, Republic of Bashkortostan, Ufa, Russian Federation; 3 Seoul National University Hospital, Cancer Research Institute, Seoul National University College of Medicine, Seoul, Republic of Korea; 4 Aix Marseille University, CNRS, INSERM, Medical Oncology Department, Institut Paoli-Calmettes, CRCM, Marseille, France; 5 Center for Breast Cancer, National Cancer Center, Goyang, Republic of Korea; 6 Centre for Experimental Cancer Medicine, Barts Cancer Institute, Queen Mary University London, London, UK; 7 National Cancer Center Hospital, Tokyo, Japan; 8 Instituto do Câncer do Estado de São Paulo, Faculdade de Medicina da Universidade do Estado do São Paulo, São Paulo, Brazil; 9 Hiroshima City Hiroshima Citizens Hospital, Hiroshima, Japan; 10 Breast Center, Department of Obstetrics and Gynecology and CCC Munich, Ludwig-Maximilians University, Munich, Germany; 11 Division of Cancer Research, Peter MacCallum Cancer Centre, Melbourne, VIC Australia; 12 The Sir Peter MacCallum Department of Medical Oncology, The University of Melbourne, Parkville, VIC Australia; 13 Department of Pathology, ZAS Hospitals, Antwerp, Belgium; 14 Merck & Co., Inc., Rahway, NJ USA; 15 Yale Cancer Center, New Haven, CT USA

**Keywords:** Breast cancer, Tumour biomarkers

## Abstract

In the randomized, phase 3 KEYNOTE-119 study, overall survival (OS) was not significantly improved with pembrolizumab 200 mg Q3W versus investigator’s choice of chemotherapy in participants with previously treated metastatic TNBC. In this exploratory analysis, we evaluated associations of tumor-infiltrating lymphocytes (TILs), T-cell‒inflamed gene expression profile (Tcell_inf_GEP), *BRCA1*/*BRCA2* mutation (BRCAm) status, homologous recombination deficiency (HRD) status, and tumor mutational burden (TMB) with clinical outcomes. TIL level was associated with improved objective response rate (ORR), progression-free survival (PFS), and OS with pembrolizumab but not with chemotherapy or after adjusting for Tcell_inf_GEP. Associations were also identified between Tcell_inf_GEP and improved ORR, PFS, and OS with pembrolizumab. Participants with TMB ≥ 10 mut/Mb showed a trend toward increased benefit with pembrolizumab versus chemotherapy. No association was seen between BRCAm/HRD status and treatment response. These findings suggest a positive association between TILs, Tcell_inf_GEP, and TMB with clinical outcomes in patients with metastatic TNBC receiving pembrolizumab. ClinicalTrials.gov Identifier: NCT02555657 (date of registration: September 18, 2015).

## Introduction

The anti–programmed cell death protein 1 (PD-1) monoclonal antibody pembrolizumab has demonstrated activity as monotherapy in certain tumor types, including in patients with advanced or metastatic disease^1^. The phase 3, randomized, open-label KEYNOTE-119 study evaluated pembrolizumab monotherapy versus physician’s choice of single-agent chemotherapy treatment as second- or third-line treatment for participants with metastatic triple-negative breast cancer (TNBC)^2^. Pembrolizumab monotherapy did not significantly improve the primary endpoint of overall survival (OS) compared with chemotherapy. However, among participants with higher programmed cell death ligand 1 (PD-L1) expression, the hazard ratio (HR) for OS favored the pembrolizumab group. Potentially, biomarkers beyond PD-L1 may be employed to identify additional patients likely to benefit from treatment with pembrolizumab monotherapy.

The presence of tumor-infiltrating lymphocytes (TILs) has been shown to be of prognostic significance in TNBC, with greater lymphocytic infiltration associated with better clinical outcomes^3,4^. In the phase 2 KEYNOTE-086 study (NCT02447003), TIL levels were a surrogate marker of preexisting antitumor immunity and were independent predictors of objective response during monotherapy with pembrolizumab^5^. Additionally, both T-cell‒inflamed gene expression profile (Tcell_inf_GEP) and *BRCA1*/*BRCA2* mutation (BRCAm) have been associated with improved treatment response in patients with TNBC^6,7^. For example, in the phase 2 KEYNOTE-086 study of pembrolizumab monotherapy for treatment of metastatic TNBC, Tcell_inf_GEP was associated with objective response rate (ORR) and OS^6^. In the phase 2 TBCRC009 study, participants with BRCAm metastatic TNBC had higher response rates to chemotherapy than those without mutations^7^. Tumor mutational burden (TMB), defined as total somatic mutations per coding area of an exon, has also been associated with improved response to treatment with immune checkpoint inhibitors, including anti–PD-1 agents, that enhance antitumor T-cell activity^8^. In a retrospective analysis, higher TMB was associated with increased response rate during treatment with anti–PD-1 or anti–PD-L1 therapy across a range of tumor types^9^. In the phase 2 KEYNOTE-158 study, participants with TMB ≥ 10 mutations/megabase (mut/Mb) had a higher ORR than participants with TMB < 10 mut/Mb (29% vs 6%), irrespective of tumor type^8^.

Additional evidence is needed to understand the ability of these biomarkers to predict response and survival outcomes with antitumor treatments such as pembrolizumab. The current analysis of data from KEYNOTE-119 evaluated associations of TILs, Tcell_inf_GEP, BRCAm/homologous recombination repair deficiency (HRD) status, and TMB as biomarkers with clinical outcomes among participants enrolled in the KEYNOTE-119 study.

## Results

### Participants

As previously reported^2^, between November 25, 2015, and April 11, 2017, 622 participants were enrolled at 150 medical centers in 31 countries and randomized to receive pembrolizumab (*n* = 312) or chemotherapy (*n* = 310). Baseline demographics for both treatment groups have been previously described^2^. In brief, among the 622 enrolled participants, 292 (46.9%) had an Eastern Cooperative Oncology Group (ECOG) performance status of 1,405 (65.1%) had PD-L1 combined positive score (CPS) ≥ 1, 194 (31.1%) had PD-L1 CPS ≥ 10, and 109 (17.5%) had PD-L1 CPS ≥ 20. The final data cutoff date was April 11, 2019. Among 601 participants in the all-patients-as-treated population (pembrolizumab, *n* = 309; chemotherapy, *n* = 292), TILs were evaluable in 551 treated participants (pembrolizumab, *n* = 284; chemotherapy, *n* = 267), Tcell_inf_GEP data were available for 333 treated participants (pembrolizumab, *n* = 177; chemotherapy, *n* = 156), BRCAm/HRD status was available for 218 treated participants (pembrolizumab, *n* = 115; chemotherapy, *n* = 103), and TMB data were available for 255 participants (pembrolizumab, *n* = 133; chemotherapy, *n* = 122).

### Association of TILs with clinical outcomes

**Fig. 1:**
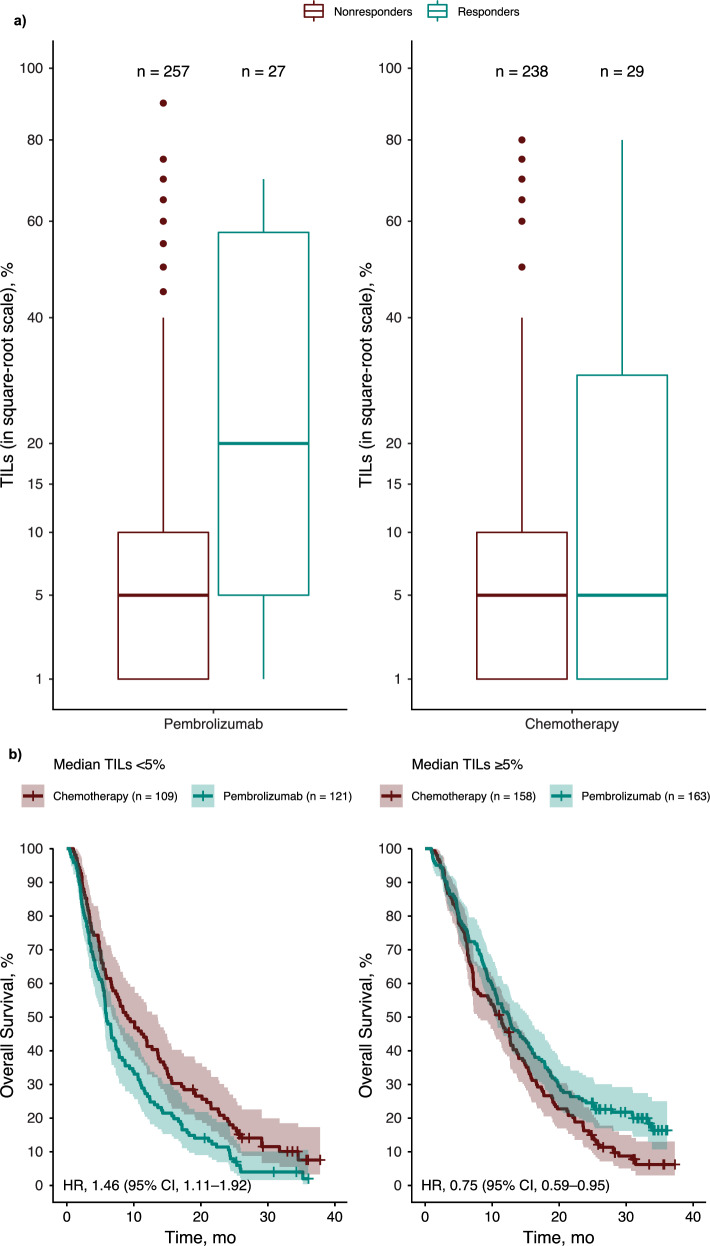
Association of TILs with treatment response. **a** TIL levels in responders and nonresponders. **b** Overall survival by median TIL cutpoints of < 5% and ≥ 5%. TILs are plotted on a square-root scale. The median TIL level was 20% among responders in the pembrolizumab group and 5% among nonresponders in the pembrolizumab group. Median TIL level was 5% among responders and nonresponders in the chemotherapy group. In these boxplots, the whiskers extend no further than 1.5 times the interquartile range; dots represent values that fall outside 1.5 times the interquartile range. Responses evaluated by blinded independent central review per RECIST version 1.1. HR hazard ratio, RECIST Response Evaluation Criteria in Solid Tumors, TIL tumor-infiltrating lymphocyte.

Across all evaluable participants, the median TIL level was 5%. ORR was 9.5% (95% CI, 6.4‒13.5%) in the pembrolizumab group (*n* = 284) and 10.9% (95% CI, 7.4‒15.2%) in the chemotherapy group (*n* = 267; Fig. 1a). Median TIL levels were higher among responders versus nonresponders in the pembrolizumab group, but not in the chemotherapy group.

In the pembrolizumab group, statistically significant associations were identified between TILs and best overall response (BOR; *P* = 0.0004), PFS (*P* = 0.0002), and OS (*P* = 0.0003). Statistical significance was not observed in the chemotherapy group for any outcome (*P* > 0.05 for all; Table 1).

**Table 1:** *P* Values^a^ for Association of TILs and Tcell_inf_GEP with Clinical Outcomes

Biomarker	Chemotherapy			Pembrolizumab		
	BOR^b^	PFS^c^	OS^c^	BOR^b^	PFS^c^	OS^c^
TILs^d^	0.1664	0.1664	0.4887	0.0004	0.0002	0.0003
Tcell_inf_GEP^e^	0.205	0.146	0.752	0.0003	8.51e-09	3.39e-07

*BOR* best overall response, *ECOG* Eastern Cooperative Oncology Group, *OS* overall survival, *PFS* progression-free survival, *Tcell*_*inf*_*GEP* T-cell‒inflamed gene expression profile, *TIL* tumor-infiltrating lymphocyte.

^a^*P* values are 1-sided for pembrolizumab and 2-sided for chemotherapy.

^b^Logistic regression model.

^c^Cox model.

^d^TILs are square-root transformed; stratification variable, previous (neo)adjuvant therapy versus de novo metastatic disease at initial diagnosis, was used as a covariate in the model for evaluating the association of TILs with clinical outcomes.

^e^The baseline ECOG performance status was used as a covariate in the model for evaluating the association of Tcell_inf_GEP with clinical outcomes.

The median TIL level of 5% was used to stratify participants into 2 groups. Among participants with TILs ≥ 5%, the HR for OS favored the pembrolizumab group (0.75 [95% CI, 0.59‒0.95]), whereas among participants with TILs < 5%, the HR for OS favored the chemotherapy group (1.46 [95% CI, 1.11‒1.92]; Fig. 1b).

Additionally, there was a moderate correlation between TIL level and PD-L1 CPS and between TIL level and Tcell_inf_GEP, with Spearman correlation coefficients of 0.46 and 0.56, respectively (Supplementary Fig. 1). Despite being correlated with one another, TIL level and PD-L1 CPS showed independent predictive ability for clinical outcomes among participants receiving pembrolizumab monotherapy in a multivariate model (TIL: BOR, *P* = 0.011; PFS, *P* = 0.003; OS, *P* = 0.004; PD-L1 CPS: BOR, *P* = 0.040; PFS, *P* = 0.038; OS, *P* = 0.090; Supplementary Table 1).

### Association of Tcell_inf_GEP with clinical outcomes

In the pembrolizumab group, statistically significant associations were identified between Tcell_inf_GEP score and BOR (*P* < 0.001), PFS (*P* < 0.0001), and OS (*P* < 0.0001). Statistical significance was not observed in the chemotherapy group for any outcome (*P* > 0.05 for all; Table 1).

Among participants with a Tcell_inf_GEP^low^ (< 1st tertile) score, the ORR was 0.0% (95% CI, 0.0‒6.8%) in the pembrolizumab group (*n* = 52) and 8.5% (95% CI, 2.8‒18.7%) in the chemotherapy group (*n* = 59). For participants with a Tcell_inf_GEP^non-low^ (≥ 1st tertile) score, the ORR was 15.2% (95% CI, 9.4‒22.7%) in the pembrolizumab group (*n* = 125) and 13.4% (95% CI, 7.3‒21.8%) in the chemotherapy group (*n* = 97; Table 2).

**Table 2:** Outcomes by Tcell_inf_GEP Status

	Low Tcell_inf_GEP (< 1st Tertile)		Non-Low Tcell_inf_GEP (≥ 1st Tertile)	
	Pembrolizumab (*n* = 52)	Chemotherapy (*n* = 59)	Pembrolizumab (*n* = 125)	Chemotherapy (*n* = 97)
Best overall response (95% CI), %	0.0 (0.0‒6.8)	8.5 (2.8‒18.7)	15.2 (9.4‒22.7)	13.4 (7.3‒21.8)
Progression-free survival				
Median (95% CI), mo	1.9 (1.6‒2.0)	3.1 (2.2‒4.3)	2.1 (2.1‒3.4)	3.6 (2.3‒4.6)
Hazard ratio (95% CI)	3.63 (2.31‒5.70)		1.40 (1.02‒1.93)	
Overall survival				
Median (95% CI), mo	5.6 (4.2‒7.0)	11.5 (7.1‒15.2)	12.7 (9.9‒15.5)	11.1 (7.3‒13.7)
Hazard ratio (95% CI)	1.72 (1.15‒2.55)		0.77 (0.58‒1.04)	

*Tcell*_*inf*_*GEP* T-cell‒inflamed gene expression profile.

**Fig. 2:**
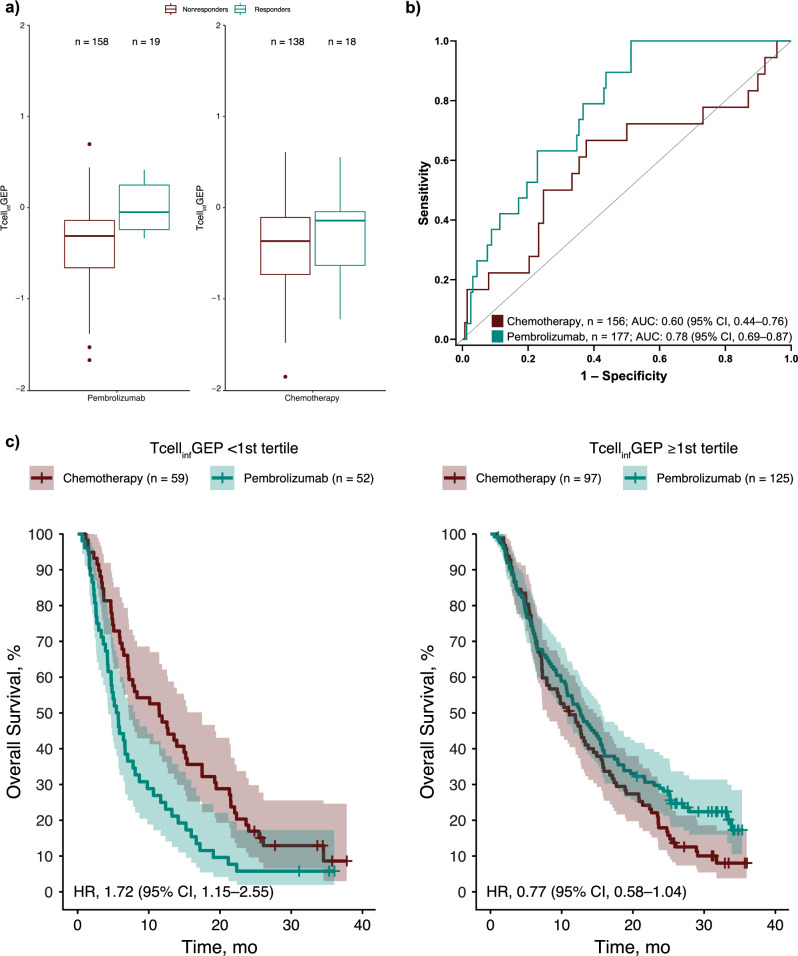
Association of Tcell_inf_GEP with treatment response. **a** Tcell_inf_GEP scores in responders and nonresponders. **b** AUROC analysis of the association between Tcell_inf_GEP score and objective response rate. **c** OS by Tcell_inf_GEP score with a 1st tertile cutpoint (< 1st tertile vs ≥ 1st tertile). In these boxplots, the whiskers extend no further than 1.5 times the interquartile range; dots represent values that fall outside 1.5 times the interquartile range. ^a^Responders include participants who had a complete or partial response per RECIST version 1.1. AUC area under the curve, AUROC area under the receiver operating characteristic curve, HR hazard ratio, RECIST Response Evaluation Criteria in Solid Tumors, Tcell_inf_GEP T-cell‒inflamed gene expression profile, TPC treatment of physician’s choice chemotherapy.

In both treatment groups, median Tcell_inf_GEP scores were higher for responders than for nonresponders, although the range of median Tcell_inf_GEP scores was narrower for the pembrolizumab group than the chemotherapy group (Fig. 2a). Area under the receiver operating characteristic curve (AUROC) analyses indicated a positive association between Tcell_inf_GEP score and ORR for the pembrolizumab group (area under the curve, 0.78 [95% CI, 0.69‒0.87]) but not for the chemotherapy group (area under the curve, 0.60 [95% CI, 0.44‒0.76]; Fig. 2b).

In the subgroup of participants with a Tcell_inf_GEP^low^ score, HRs for PFS and OS favored the chemotherapy group. The HR for PFS was 3.63 (95% CI, 2.31‒5.70) and the HR for OS was 1.72 (95% CI, 1.15‒2.55; Table 2). In the subgroup of participants with Tcell_inf_GEP^non-low^, the HR for PFS was 1.40 (95% CI, 1.02‒1.93) and the HR for OS was 0.77 (95% CI, 0.58‒1.04; Fig. 2c).

Additional analyses were done to model associations jointly of Tcell_inf_GEP and TILs with clinical outcomes. In the pembrolizumab group, 165 participants had both Tcell_inf_GEP and TIL data available for analysis; in the chemotherapy group, 146 participants had both Tcell_inf_GEP and TIL data available. When Tcell_inf_GEP was modeled jointly with TILs (square-root transformed), statistically significant associations were identified for the pembrolizumab group between Tcell_inf_GEP score and BOR (*P* = 0.006), PFS (*P* < 0.001), and OS (*P* < 0.001), but no statistically significant associations were identified for the chemotherapy group (*P* > 0.05 for all). However, when TILs were modeled jointly with Tcell_inf_GEP score, no association was identified between TILs and BOR, PFS, or OS for either treatment group (*P* > 0.15 for all; Table 3).

**Table 3:** *P* Values for Association of TILs and Tcell_inf_GEP with Clinical Outcomes Using a Multivariate Model

Biomarker	Chemotherapy				Pembrolizumab			
	N	BOR^a^	PFS^b^	OS^b^	N	BOR^a^	PFS^b^	OS^b^
Tcell_inf_GEP	146	0.788	0.384	0.760	165	0.006_+_	3.83e-05_+_	3.38e-04_+_
TILs^c^	146	0.182	0.710	0.938	165	0.461	0.297	0.198

*BOR* best overall response, *OS* overall survival, *PFS* progression-free survival, *Tcell*_*inf*_*GEP* T-cell‒inflamed gene expression profile, *TIL* tumor-infiltrating lymphocyte.

^a^BOR: Logistic regression model.

^b^PFS and OS: Cox model. All models include the baseline ECOG performance status as a covariate.

^c^TILs are square-root transformed.

+: indicates the observed association is positive.

There was a moderate correlation between Tcell_inf_GEP and PD-L1 CPS with a Spearman correlation coefficient of 0.6 (Supplementary Fig. 1).

### Association of BRCAm/HRD status with clinical outcomes

Among 218 participants with evaluable data, 78 of 115 (67.8%) in the pembrolizumab group and 64 of 103 (62.1%) in the chemotherapy group were assessed as BRCAm or HRD-positive (genomic loss of heterozygosity [gLOH] ≥ 16). Forty-five participants had BRCAm (pembrolizumab, *n* = 26; chemotherapy, *n* = 19) and 128 had HRD-positive (gLOH ≥ 16; pembrolizumab, *n* = 69; chemotherapy, *n* = 59) status. No statistically significant association was observed in either treatment arm among the 3 BRCAm/HRD status categories and BOR, PFS, or OS (*P* > 0.05 for all).

### Association of TMB with clinical outcomes

**Fig. 3:**
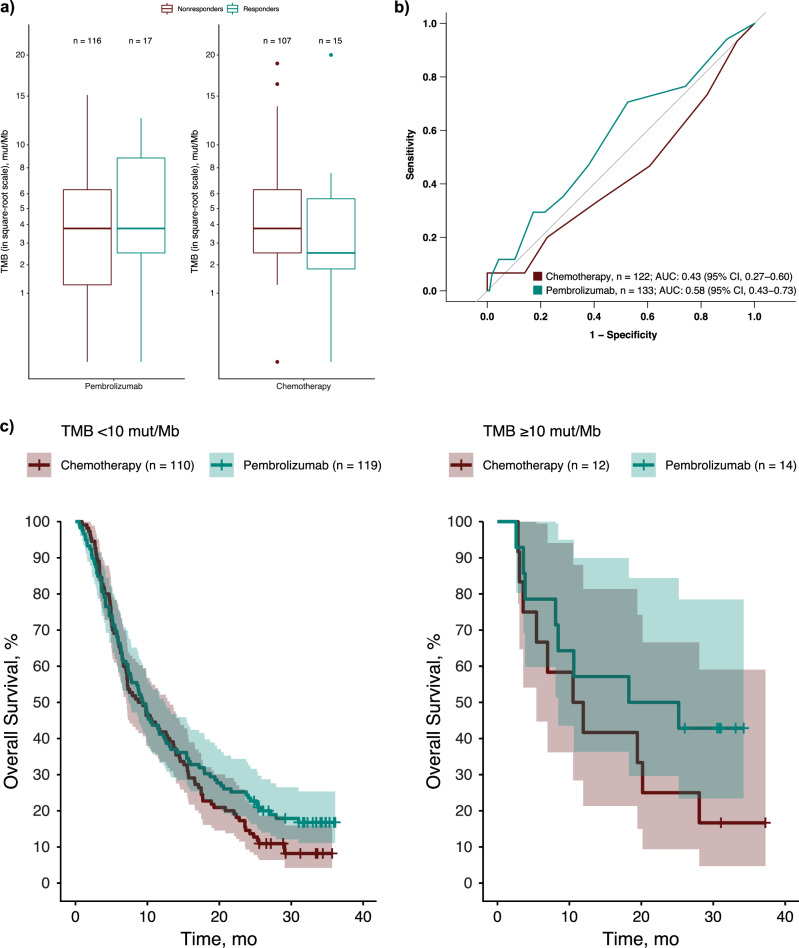
Association of TMB with treatment response. **a** TMB in responders and nonresponders. **b** AUROC analysis of the association between TMB and objective response rate. **c** Overall survival in participants with TMB ≥ 10 mut/Mb and participants with TMB < 10 mut/Mb. Median TMB was 3.78 mut/Mb among both responders and nonresponders in the pembrolizumab group; median TMB was 2.52 mut/Mb among responders in the chemotherapy group and 3.78 mut/Mb among responders in the chemotherapy group. In these boxplots, the whiskers extend no further than 1.5 times the interquartile range; dots represent values that fall outside 1.5 times the interquartile range. ^a^Responders include participants who had a complete or partial response per RECIST version 1.1. AUROC area under the receiver operating characteristic curve, mut/Mb mutations per megabase, RECIST Response Evaluation Criteria in Solid Tumors, TMB tumor mutational burden.

Overall, 26 of 255 participants (10.2%) had TMB ≥ 10 mut/Mb assessed using the FoundationOne^®^CDx comprehensive genomic profiling assay (Foundation Medicine, Inc., Cambridge, MA, USA). Among all participants, though small in numbers, the association between higher TMB and better clinical outcomes was statistically significant for PFS (*P* = 0.015) and OS (*P* = 0.025) but not BOR (*P* = 0.154) with pembrolizumab. Median TMB was similar among responders versus nonresponders in the pembrolizumab group (albeit with a higher interquartile range for responders), whereas the median TMB was slightly lower among responders versus nonresponders in the chemotherapy group (Fig. 3a). The AUROC for the association between TMB and ORR was 0.58 (95% CI, 0.43‒0.73) for pembrolizumab and 0.43 (95% CI, 0.27‒0.60) for chemotherapy (Fig. 3b).

There was no association between TMB and ORR, PFS, or OS with pembrolizumab or with chemotherapy (*P* > 0.05 for all). OS was longer for participants with TMB ≥ 10 mut/Mb treated with pembrolizumab than for all other groups (Fig. 3c).

## Discussion

In these exploratory analyses from the phase 3 KEYNOTE-119 study of pembrolizumab monotherapy versus chemotherapy in participants with metastatic TNBC, we evaluated associations between clinical outcomes and a range of potential biomarkers of response to treatment. We identified important associations between these biomarkers and ORR, PFS, and OS among participants receiving pembrolizumab monotherapy; these results may inform the design of future studies evaluating pembrolizumab treatment in patients with advanced or metastatic TNBC.

In the exploratory analysis of TIL expression and clinical outcomes, TIL ≥ 5% was significantly associated with better ORR, PFS, and OS with pembrolizumab monotherapy but not with single-agent chemotherapy. As with CPS, efficacy estimates at the median TIL cutpoint (≥ 5%) suggest that a subset of patients with metastatic TNBC necessitating later-line therapy can derive prolonged survival benefit from pembrolizumab over chemotherapy. Although TIL expression and CPS were moderately correlated, they were independently predictive of clinical outcomes. Understanding which patients with later-line metastatic TNBC would benefit from pembrolizumab would need to be tested in a prospective study; however, TIL expression represents a pragmatic and globally accessible technique that seems to evaluate patients for a pre-existing immune response.

Findings from exploratory analyses also suggest a positive association between Tcell_inf_GEP score and objective response, PFS, and OS for patients with metastatic TNBC who were treated with pembrolizumab, but not in those treated with chemotherapy. A more favorable treatment effect was observed for PFS and OS in the Tcell_inf_GEP-enriched population. Our results are consistent with those from other studies that have evaluated associations between Tcell_inf_GEP score and clinical outcomes among patients receiving pembrolizumab. In particular, in the phase 2 KEYNOTE-086 study, which evaluated pembrolizumab monotherapy in participants with metastatic TNBC who received 1 or 2 prior lines of therapy, statistically significant associations between a higher Tcell_inf_GEP score and improved ORR and OS were identified^6^. Statistically significant associations between Tcell_inf_GEP score and improved clinical outcomes with pembrolizumab treatment have also been identified for participants with advanced urothelial carcinoma^10^, head and neck squamous cell carcinoma^11^, advanced gastric cancer (in KEYNOTE-061)^12^, and non‒small-cell lung cancer^13^. The current results, taken together with prior findings in other tumor types, support the association of Tcell_inf_GEP score with response to pembrolizumab treatment across multiple tumor types.

The 18 genes that comprise Tcell_inf_GEP were identified based on their association with response among 1188 participants receiving pembrolizumab in clinical trials^14^. Results from our study support the hypothesis that increased expression of genes associated with T-cell activation and recruitment within the tumor microenvironment may play a key role in modulating response to immune checkpoint blockade in metastatic TNBC. However, at present, the mechanisms mediating associations between tumor inflammatory pathways assessed by Tcell_inf_GEP score and outcomes in patients with metastatic breast cancer are not fully understood and require further elucidation. Notably, we observed that the associations between Tcell_inf_GEP score and efficacy outcomes were independent of TILs, as determined by joint modeling, which have previously been shown to be associated with response to pembrolizumab (but not chemotherapy), as demonstrated in participants enrolled in the KEYNOTE-119 trial^6^. Importantly, after adjusting for Tcell_inf_GEP score, the association between TILs and clinical response to pembrolizumab was no longer statistically significant. Taken together, these findings suggest a potential quantitative and biologic relationship between TILs and Tcell_inf_GEP, where TILs do not provide additional predictive value beyond that provided by Tcell_inf_GEP. Further studies will be needed to elucidate the nature of such a relationship.

No association was found between BRCAm/HRD status and clinical outcomes in either treatment group. To our knowledge, 2 other studies have evaluated the association between BRCAm/HRD status and clinical outcomes in advanced solid tumors treated with pembrolizumab. In the phase 2 TOPACIO study of niraparib plus pembrolizumab in women with advanced or metastatic TNBC, the ORR was 47% for participants with BRCAm tumors and 11% for those without confirmed tumor BRCAm^15^. Median PFS was 8.3 and 2.1 months, respectively. Similarly, initial results from the phase 2 KEYLYNK-007 study of olaparib plus pembrolizumab in advanced solid tumors showed an ORR of 29% for participants with BRCAm tumors and 21% for participants with HRD-positive tumors. Confirmed responses were reported for participants with breast, cervical, endometrial, prostate, duodenal, ovarian, peritoneal, and non‒small-cell lung cancers. The duration of response was not reached for both participant subgroups; the median PFS was not reached in the BRCAm subgroup and 4.1 months in the HRD-positive subgroup^16^. Yet, since pembrolizumab was combined with a PARP inhibitor in both studies, no clear conclusion could be drawn.

In the exploratory analysis of TMB and clinical outcomes, there was a trend toward a positive association between TMB and clinical response with pembrolizumab but not with chemotherapy. Although the association between TMB and ORR was not statistically significant, those with PFS and OS were. Notably, OS was longer for participants with TMB ≥ 10 mut/Mb with a clear and consistent separation of Kaplan-Meier curves for OS, and a similar trend was also observed in participants with TMB < 10 mut/Mb, albeit less pronounced. However, it must be noted that there were relatively few participants with TMB ≥ 10 mut/Mb (10.2%), which may have limited the ability to assess the predictive value of TMB in this setting. Our findings are broadly consistent with those from the KEYNOTE-158 study, in which TMB ≥ 10 mut/Mb was associated with response to pembrolizumab among participants with advanced solid tumors (none of whom had breast cancer)^8^.

Other studies have evaluated biomarkers for immunotherapy in patients with TNBC and provide important context for our findings. The phase 3 IMpassion130 study showed a clinical benefit with atezolizumab (an anti–PD-L1 antibody) plus chemotherapy versus chemotherapy alone in patients with locally advanced or metastatic TNBC with PD-L1 expression on tumor-infiltrating immune cells (PD-L1 IC+) but not in the intent-to-treat population (ie, irrespective of PD-L1 IC status)^17^. In exploratory analyses from IMpassion130^18^, tumor CD8 positivity and stromal TIL positivity were associated with improved outcomes. However, these biomarkers were highly correlated with PD-L1 IC+ and improvements in outcomes were confined to patients who were also PD-L1 IC+, suggesting these biomarkers did not add predictive value beyond PD-L1 status. In the ALICE study (a phase 2b trial evaluating the addition of atezolizumab to anthracycline-based chemotherapy in patients with metastatic TNBC), there was no evidence of an association between PD-L1 status and PFS or between TMB and PFS among patients receiving atezolizumab plus chemotherapy; however, in exploratory analyses, there was evidence of greater PFS benefit among patients with an above-median tumor inflammation signature^19^. In a combined analysis of circulating tumor cells (CTCs) from patients in the ALICE study and the ICON study (a phase 2b trial evaluating immunogenic chemotherapy combined with ipilimumab and nivolumab in patients with metastatic hormone receptor positive HER2− breast cancer), presence of CTCs was associated with poor prognosis: 6 out of 17 CTC-positive patients showed PD-L1 expression, which was associated with significantly shorter OS^20^. Results from the single-arm TONIC study (a phase 2 trial evaluating efficacy of nivolumab after short-term induction with low-dose chemotherapy in patients with metastatic TNBC) showed that patients who exhibited clinical benefit had higher levels of stromal TILs and higher levels of CD8 and PD-L1 expression on immune cells^21^. Finally, results from the SAFIR02-BREAST IMMUNO study showed that, among patients with metastatic TNBC durvalumab versus maintenance therapy, there was no association between OS and tumor infiltration by lymphocytes, although there was an association between OS and CD274 amplification/gain and efficacy^22^.

A range of novel biomarkers are currently being evaluated for their potential to predict response to immunotherapy in patients with breast cancer. Emerging evidence has suggested there may be associations between efficacy outcomes and potential biomarkers, including gene-signature scores for T helper 1 cells, B cells, and neutrophils^21^, and gut microbiota diversity^23^.

A key strength of the current findings is that they are based on data from a randomized, phase 3 trial. The presence of a comparator group allowed us to demonstrate that the association between TILs and Tcell_inf_GEP scores and outcomes was only observed in participants receiving pembrolizumab. An additional strength of the analysis is the large size of the biomarker-evaluable populations; among the 601 participants enrolled and treated in KEYNOTE-119, approximately a little more than half in both treatment arms had Tcell_inf_GEP data, and a little more than one-third of participants had BRCAm/HRD data available for analysis. However, these descriptive results should be interpreted with caution due to the retrospective, exploratory nature of the analysis and the small numbers of participants in several of the subgroups. In addition, the tissue samples reflect a mix of newly obtained (~56%) and archival (~39%) samples collected at different times throughout a participant’s treatment history, which may have affected biomarker analysis^24^.

In summary, Tcell_inf_GEP score, TIL quantity, and TMB status may be predictive of treatment response to pembrolizumab among patients with metastatic TNBC. Further investigation will be needed to ascertain how these biomarkers may be used along with PD-L1 immunohistochemistry to ensure all patients who will benefit from pembrolizumab can receive it.

## Methods

### Eligibility criteria

Full eligibility criteria and study design have been published previously^2^. Briefly, participants with centrally confirmed metastatic TNBC were eligible if they had an ECOG performance status of 0 or 1; had received 1 or 2 prior systemic treatments for metastatic breast cancer; had received prior treatment with an anthracycline or taxane in the neoadjuvant, adjuvant, or metastatic setting; had documented disease progression on the most recent therapy; and had measurable disease per Response Evaluation Criteria in Solid Tumors (RECIST) version 1.1. Participants were excluded if they had active central nervous system metastases or carcinomatous meningitis, active autoimmune disease requiring systemic treatment in the past 2 years, or history or evidence of noninfectious pneumonitis requiring steroid treatment; had received treatment with an agent directed at PD-1, PD-L1, or another coinhibitory T-cell receptor or with an antineoplastic monoclonal antibody within 4 weeks; or had received chemotherapy, targeted small-molecule therapy, or radiotherapy within 2 weeks. Participants were required to provide a newly obtained tumor tissue sample for central confirmation of TNBC status and PD-L1 expression; archival samples were permitted only with approval from the study sponsor if a new sample could not be obtained.

The study was done in accordance with the principles set forth in the Declaration of Helsinki and with the International Council on Harmonisation Good Clinical Practice guidelines. An independent ethics committee or institutional review board at each site (listed in the Supplementary Materials) approved the protocol before study-related procedures began. All participants provided written informed consent before enrolling. The study was registered with ClinicalTrials.gov (NCT02555657; date of registration: September 18, 2015).

### Study treatment

Briefly, participants were randomized 1:1 to receive pembrolizumab or chemotherapy. Randomization was stratified by tumor PD-L1 status (CPS ≥ 1 vs CPS < 1) and by prior neoadjuvant or adjuvant treatment versus de novo metastatic TNBC. Participants randomized to the pembrolizumab group received pembrolizumab 200 mg once every 3 weeks for 35 cycles or until confirmed disease progression, unacceptable toxicity, consent withdrawal, or investigator decision. Participants randomized to the chemotherapy group received the investigator’s choice of capecitabine, eribulin, gemcitabine, or vinorelbine per local guidelines until disease progression, unacceptable toxicity, consent withdrawal, or investigator decision. Participants in the chemotherapy group who had documented disease progression were not permitted to cross over to receive pembrolizumab.

### Assessments

The presence of TILs in tumor samples was assessed by light microscopy of hematoxylin and eosin‒stained sections using a predefined method^25,26^. Tumor RNA profiling was conducted on the RNA-seq platform (Illumina, San Diego, CA). TMB, BRCAm, and HRD status were assessed using the FoundationOne^®^CDx comprehensive genomic profiling assay (Foundation Medicine, Inc., Cambridge, MA, USA)^27–29^. FoundationOne^®^CDx is an FDA-approved next-generation sequencing assay that can identify TMB status in tumor samples using a prespecified cutpoint of at least 10 mut/Mb to define TMB-high (≥ 10 mut/Mb) and less than 10 mut/Mb for non–TMB-high (< 10 mut/Mb) tumors^8^.

BRCAm/HRD status was categorized into 3 groups: BRCAm, *BRCA* wild-type with gLOH ≥ 16, and *BRCA* wild-type with gLOH < 16.

Tumor imaging was performed using computed tomography or magnetic resonance imaging at baseline, every 9 weeks for 1 year, and every 12 weeks thereafter until participants had radiologic evidence of disease progression, started a new anticancer treatment, or withdrew consent, or until the end of the study. Tumor response was assessed by a central imaging vendor per RECIST version 1.1.

### Statistical analyses

The study’s primary endpoint was OS (defined as time from randomization to death from any cause). ORR and PFS (time from randomization to disease progression or death) were secondary endpoints. Evaluation of the relationships between potential biomarkers and antitumor activity of pembrolizumab was an exploratory endpoint.

Biomarker analyses were performed in the all-patients-as-treated population (ie, those who received ≥ 1 dose of study medication analyzed according to treatment received). This exploratory analysis assessed the associations of TILs, Tcell_inf_GEP, BRCAm/HRD status, and TMB with the clinical outcomes of BOR and PFS per RECIST version 1.1 and with OS. The associations of biomarkers with BOR were evaluated using logistic regression and AUROC analysis; their associations with PFS and OS were evaluated by Cox proportional hazards models. Associations were evaluated within each treatment group (ie, for participants in the pembrolizumab group and participants in the chemotherapy group). Analyses for Tcell_inf_GEP, TMB, and BRCAm/HRD were adjusted for baseline ECOG performance status, and analyses of TILs were adjusted for previous adjuvant or neoadjuvant therapy versus de novo metastatic disease at initial diagnosis.

Relative prognostic and predictive effects of the biomarkers were assessed in subgroups defined as follows: above or below the median TIL score; Tcell_inf_GEP^low^ (< 1st tertile) versus Tcell_inf_GEP^non-low^ (≥ 1st tertile); BRCAm/HRD status (BRCAm vs *BRCA* wild-type and gLOH ≥ 16 vs *BRCA* wild-type and gLOH < 16); and TMB ≥ 10 mut/Mb versus TMB < 10 mut/Mb. The cutoff for dichotomization of the population by Tcell_inf_GEP (ie, < 1st tertile/≥ 1st tertile) was selected based on prior studies evaluating Tcell_inf_GEP as a biomarker for pembrolizumab^30,31^.

The 95% CIs for ORRs were estimated using the Clopper and Pearson method^32^. The 95% CIs for HRs from Cox proportional hazards models were calculated using the log-log method with adjustment for baseline ECOG performance status. No alpha was assigned to these analyses; all *P* values are descriptive. In multivariate modeling, Tcell_inf_GEP and TILs were jointly modeled with baseline ECOG performance status as a covariate; CPS and TILs were jointly modeled with the stratification variables, previous adjuvant or neoadjuvant therapy versus de novo metastatic disease at initial diagnosis, as covariates. Spearman correlation (ρ) was used to assess the pairwise correlations between Tcell_inf_GEP, PD-L1 CPS, TILs, and TMB.

## Supplementary information

Supplementary Materials

## Data Availability

Merck Sharp & Dohme LLC, a subsidiary of Merck & Co., Inc., Rahway, NJ, USA (MSD) is committed to providing qualified scientific researchers access to anonymized data and clinical study reports from the company’s clinical trials for the purpose of conducting legitimate scientific research. MSD is also obligated to protect the rights and privacy of trial participants and, as such, has a procedure in place for evaluating and fulfilling requests for sharing company clinical trial data with qualified external scientific researchers. The MSD data sharing website (available at: https://externaldatasharing-msd.com/) outlines the process and requirements for submitting a data request. Applications will be promptly assessed for completeness and policy compliance. Feasible requests will be reviewed by a committee of MSD subject matter experts to assess the scientific validity of the request and the qualifications of the requestors. In line with data privacy legislation, submitters of approved requests must enter into a standard data-sharing agreement with MSD before data access is granted. Data will be made available for request after product approval in the US and EU or after product development is discontinued. There are circumstances that may prevent MSD from sharing requested data, including country- or region-specific regulations. If the request is declined, it will be communicated to the investigator. Access to genetic or exploratory biomarker data requires a detailed, hypothesis-driven statistical analysis plan that is collaboratively developed by the requestor and MSD subject matter experts; after approval of the statistical analysis plan and execution of a data-sharing agreement, MSD will either perform the proposed analyses and share the results with the requestor or will construct biomarker covariates and add them to a file with clinical data that is uploaded to an analysis portal so that the requestor can perform the proposed analyses.
