# Supplementary material for: Association of potential biomarkers with clinical outcomes in metastatic triple-negative breast cancer treated with pembrolizumab or chemotherapy
Source: NPJ Breast Cancer. 2025 Oct 2;11:109. doi: 10.1038/s41523-025-00814-y (PMC12491503; doi:10.1038/s41523-025-00814-y)
Supplement: Supplementary file 1 — Supplementary Materials [file 41523_2025_814_MOESM1_ESM.docx]

# Supplementary Materials

# Supplementary Table 1. *P* Values^a^ for Association of TIL Level and PD-L1 CPS With Clinical Outcomes Using a Multivariate Model

| **Treatment Arm** | **Variable^b^** | **BOR** | **PFS** | **OS** |
| --- | --- | --- | --- | --- |
| Pembrolizumab | TILs | 0.011 | 0.003 | 0.004 |
|  | CPS | 0.040 | 0.038 | 0.090 |
| Chemotherapy | TILs | 0.150 | 0.051 | 0.286 |
|  | CPS | 0.652 | 0.027 | 0.264 |

BOR, best overall response; CPS, combined positive score; PFS, progression-free survival; OS, overall survival; PD-L1, programmed cell death ligand 1; TIL, tumor-infiltrating lymphocyte.

Stratification variable: previous (neo)adjuvant therapy versus de novo metastatic disease at initial diagnosis was used as a covariate in the model.

^a^*P* values are 1-sided for pembrolizumab and 2-sided for chemotherapy.

^b^TILs and CPS are both square-root transformed.

# **Supplementary Figure 1.** Correlations between analyzed biomarkers. CPS, combined positive score; PD-L1, programmed cell death ligand 1; Tcell_inf_GEP, T-cell‒inflamed gene expression profile; TIL, tumor-infiltrating lymphocyte; TMB, tumor mutational burden.


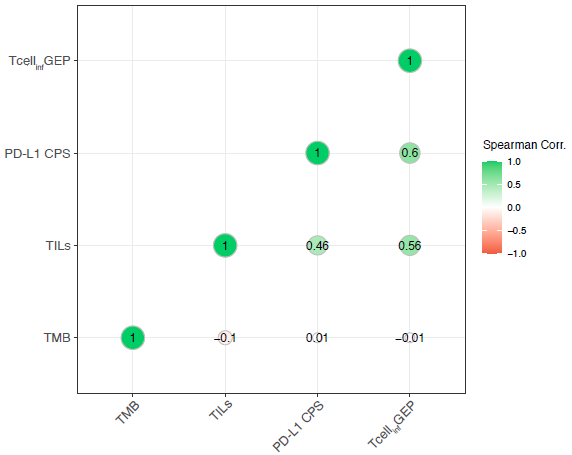


# Names of ethics bodies that approved the trial protocol

| **Country** | **Site Name** | **Investigator Name** | **Participants, n** | **Ethics Body** |
| --- | --- | --- | --- | --- |
| Russia | Republican Clinical Oncology Dispensary of Republic of Bashkortostan | Lipatov, Oleg | 19 | LEC of Republican Clinical Oncology Dispensary |
| South Korea | Seoul National University Hospital | Im, Seock-Ah | 15 | Seoul National University Hospital IRB/IEC |
| France | Institut Paoli Calmettes | Goncalves, Anthony | 14 | CPP "Sud Est III" |
| Spain | Hospital Vall D Hebron | Munoz Couselo, Eva | 14 | Hospital Vall D Hebron |
| Poland | Centrum Onkologii Instytut im. Marii Skłodowskiej Curie | Nowecki, Zbigniew | 12 | Centrum Onkologii-Instytut im. Marii Skłodowskiej-Curie |
| U.K. | Barts Cancer Institute | Schmid, Peter | 12 | London-Bloomsbury Research Ethics Committee |
| South Korea | National Cancer Center | Lee, Keun Seok | 12 | National Cancer Center IRB/IEC |
| Singapore | National Cancer Centre Singapore | Dent, Rebecca | 11 | National Cancer Centre IRB |
| Japan | National Cancer Center Hospital | Tamura, Kenji | 11 | National Cancer Center Institutional  Review Board |
| Germany | Universitaetsklinikum Hamburg Eppendorf | Witzel, Isabell | 10 | Universitaet Erlangen Nuernberg |
| U.K. | Royal Marsden Hospital | Turner, Nicholas | 10 | London-Bloomsbury Research Ethics Committee |
| Brazil | Instituto do Cancer de Sao Paulo - ICESP | Testa, Laura | 10 | Comite de Etica em Pesquisa da Faculdade de Medicina da Universidade de Sao Paulo  CONEP - Comissao Nacional de Etica em Pesquisa |
| Japan | Hiroshima City Hiroshima Citizens Hospital | Ohtani, Shoichiro | 10 | Hiroshima City Hiroshima Citizens Hospital Institutional Review Board |
| Italy | Ospedale San Raffaele | Gianni, Luca | 9 | Comitato Etico Ospedale San Raffaele |
| Mexico | Consultorio de Medicina Especializada del Sector Privado | Juarez Ramiro, Alejandro | 9 | Comite de Etica en Investigacion de  Mexico Centre for Clinical Research S.A. de C.V. CBIC |
| France | Centre Leon Berard | Tredan, Olivier | 8 | CPP "Sud Est III" |
| Germany | Klinikum der Universit. Muenchen | Harbeck, Nadia | 8 | Universitaet Erlangen Nuernberg |
| Italy | IRCCS Istituto Oncologico Veneto | Guarneri, Valentina | 8 | IRCCS Istituto Oncologico Veneto IRB/IEC |
| Russia | Russian Oncological Research Center n.a. N.N.Blokhin of MoH | Lichinitser, Mikhail Romanovich/  Gutorov, Sergey | 8 | N.N. Blokhin NMRCO IRB/IEC |
| Brazil | Clinica de Pesquisas e Centro de Estudos Onc. Ginecol. e Mamaria Ltda | Hegg, Roberto | 8 | Comite de Etica em Pesquisa do Centro de Referencia da Saude da Mulher  CONEP - Comissao Nacional de Etica  em Pesquisa |
| Mexico | Centro de Investigacion y Transferencia en Salud | Martinez Rodriguez, Jorge Luis | 8 | Comite Etica Investigacion Escuela Medicina Instituto Tecnologico y Estudios Superiores Monterrey |
| Australia | Hollywood Private Hospital | Chan, Arlene | 8 | Bellberry Human Resources Committee |
| Japan | St.Luke's International Hospital | Yamauchi, Teruo | 8 | St.Luke's International Hospital Institutional Review Board |
| France | Institut Gustave Roussy | Andre, Fabrice | 7 | CPP "Sud Est III" |
| Spain | Hospital General Universitario  Ramon y Cajal | Cortes, Javier | 7 | Hospital Vall D Hebron |
| Ireland | Mater Misericordiae University Hospital | Kelly, Catherine Margaret | 7 | Clinical Research Ethics Committee of the Cork Teaching Hospitals |
| U.K. | The Beatson West of Scotland Cancer Centre | MacPherson, Iain | 7 | London-Bloomsbury Research Ethics Committee |
| Malaysia | Pantai Hospital Kuala Lumpur | Yusof, Mastura Md | 7 | Medical Ethics & Research  Committee (MREC) |
| U.S.A. | Dana Farber Cancer Institute | Winer, Eric | 6 | Dana Farber Cancer Institute Institutional Review Board |
| U.S.A. | Cleveland Clinic | Montero, Albert | 6 | Cleveland Clinic Foundation |
| Germany | Universitaets-Frauenklinik Tuebingen | Grischke, Eva-Maria | 6 | Universitaet Erlangen Nuernberg |
| Italy | Istituto Europeo di Oncologia | Colleoni, Marco | 6 | Istituto Europeo di Oncologia |
| Poland | Centrum Onkologii im prof Franciszka Lukaszczyka | Chmielowska, Ewa | 6 | Centrum Onkologii-Instytut im. Marii Skłodowskiej-Curie |
| Turkey | Trakya Univ. Tip Fakultesi | Cicin, Irfan | 6 | Ege Uni. Tip Fakultesi Arastirma Etik Kurulu |
| Turkey | Acibadem Adana Hastanesi | Abali, Huseyin/ Yavuz, Sinan | 6 | Ege Uni. Tip Fakultesi Arastirma Etik Kurulu |
| Turkey | Ege Universitesi Tıp Fakultesi | Uslu, Ruchan F./Gokmen, Erhan | 6 | Ege Uni. Tip Fakultesi Arastirma Etik Kurulu |
| Brazil | Hospital Sao Jose | Andrade, Juliana Pimenta | 6 | Comite de Etica em Pesquisa da Real e Benemerita Associacao Portuguesa de Beneficencia  CONEP - Comissao Nacional de Etica  em Pesquisa |
| Australia | Southern Medical Day Care Centre | Aghmesheh, Morteza/  Tafreshi, Ali | 6 | Bellberry Limited |
| Philippines | St. Luke’s Medical Center | Li, Rubi K. | 6 | St. Luke s Medical Center IRB/IEC |
| Taiwan | Chang Gung Memorial Hospital | Chen, Shin-Cheh | 6 | Chang Gung Medical Foundation Institutional Review Board A |
| Japan | Hyogo College of Medicine Hospital | Miyoshi, Yasuo | 6 | Hyogo College of Medicine Hospital Institutional Review Board |
| Japan | Saitama Cancer Center | Inoue, Kenichi | 6 | Saitama Cancer Center Institutional Review Board |
| Japan | Chiba Cancer Center | Yamamoto, Naohito | 6 | Chiba Cancer Center Institutional Review Board |
| U.S.A. | Magee Women's Hospital | Brufsky, Adam | 5 | Western Institutional Review Board |
| Germany | Universitat Erlangen-Nurnberg | Fasching, Peter A. | 5 | Universitaet Erlangen Nuernberg |
| Italy | Istituto Nazionale dei Tumori Regina Elena IRCCS - IFO | Fabi, Alessandra | 5 | Istittuto Nazionale dei Tumori Regina  Elena IRCCS - IFO |
| Mexico | Grupo Medico Camino SC | Garnica Jaliffe, Georgina | 5 | Comite de Etica en Investigacion del Grupo Medico Camino SC |
| South Korea | Korea University Anam Hospital | Park, Kyong Hwa | 5 | Korea University Anam Hospital IRB |
| Japan | Shizuoka Cancer Center | Watanabe, Junichiro | 5 | Shizuoka Cancer Center Hospital and Research Institute IRB |
| Japan | The Cancer Institute Hospital of JFCR | Ito, Yoshinori | 5 | The Cancer Institute Hospital of JFCR Institutional Review Board |
| U.S.A. | The West Clinic | Vidal, Gregory | 4 | Western Institutional Review Board |
| U.S.A. | Memorial Sloan Kettering Cancer Center | McArthur, Heather/ Gucalp, Ayca | 4 | Memorial Sloan Kettering Cancer Center IRB |
| U.S.A. | Virginia Oncology Associates | Danso, Michael A. | 4 | US Oncology, Inc. IRB |
| Belgium | Universitair Ziekenhuis Brussel | Fontaine, Christel | 4 | Universitair Ziekenhuis Brussel - Commissie Medische Ethiek |
| France | Institut Claudius Regaud | Dalenc, Florence | 4 | CPP "Sud Est III" |
| U.K. | Royal Marsden Hospital | Turner, Nicholas | 4 | London-Bloomsbury Research Ethics Committee |
| U.K. | St James’s Institute of Oncology | Twelves, Christopher J. | 4 | London-Bloomsbury Research Ethics  Committee |
| Russia | Republican Clinical Oncology Dispensary of Tatarstan MoH | Mukhametshina, Guzel Zinnurovna | 4 | LEC of Tatarstan Republican Clinical  Oncology Dispensary |
| Russia | Saint Petersburg SBHI City Clinical Oncological Dispensary | Manikhas, Alexey Georgievich | 4 | SPb SBHI City Clinical Oncological Dispensary IRB/IEC |
| Peru | Clinica Montecarmelo | Alvarez, Renzo | 4 | Comite de Etica Asociación Benefica  Prisma |
| Taiwan | National Cheng Kung University Hospital | Su, Wu-Chou | 4 | National Cheng Kung University Hospital |
| Japan | National Cancer Center Hospital East | Mukai,  Hirofumi | 4 | National Cancer Center Institutional  Review Board |
| Japan | National Hospital Organization Hokkaido Cancer Center | Takahashi, Masato | 4 | National Hospital Organization Hokkaido Cancer Center IRB |
| Japan | Saitama Medical University International Medical Center | Saeki, Toshiaki/ Osaki, Akihiko | 4 | Saitama Medical University International Medical Center IRB |
| Japan | Kindai University Hospital | Tsurutani, Junji/ Iwasa, Tsutomu | 4 | Kindai University Hospital Institutional Review Board |
| U.S.A. | Montefiore-Einstein Center | Sparano, Joseph A. | 3 | Biomedical Research Alliance of New York IRB |
| U.S.A. | Vanderbilt Health One | Abramson, Vandana G. | 3 | Vanderbilt University Institutional Review Board |
| U.S.A. | OHSU Center for Health & Healing | Kemmer, Kathleen/Mitri, Zahi | 3 | OHSU Institutional Review Board |
| Germany | Gynaekologisches Zentrum-  Schwerpunkt Gyn. Onkologie | Kurbacher, Christian Martin | 3 | Universitaet Erlangen Nuernberg |
| Netherlands | Universitair Medisch Centrum Utrecht | Suijkerbuijk, Karijn | 3 | Stichting BEBO |
| Poland | Centrum Onkologii Ziemii Lubelskiej | Kukielka-Budny, Bozena | 3 | Centrum Onkologii-Instytut im. Marii Skłodowskiej-Curie |
| Poland | Uniwersyteckie Centrum Kliniczne | Jassem, Jacek | 3 | Centrum Onkologii-Instytut im. Marii  Skłodowskiej-Curie |
| Spain | Hospital General de Elche | Rodriguez-Lescure, Alvaro | 3 | Hospital Vall D Hebron |
| Spain | Hospital General Universitario Gregorio Maranon | Martin Jimenez, Miguel | 3 | Hospital Vall D Hebron |
| Sweden | Onkologi Kliniken | Lindman, Henrik | 3 | Regionala etikprovningsnamnden |
| Hong Kong | Queen Mary Hospital | Wing Yan, Joanne Chiu | 3 | HKU/HA HK West Cluster Institutional Review Board (HKU/HA  HKW IRB) |
| Russia | Russian Oncological Research Center n.a. N.N.Blokhin of MoH | Tjulandin, Sergey | 3 | EC of RORC n.a. N.N. Blokhin RAMS  N.N. Blokhin NMRCO IRB/IEC |
| Russia | St. Petersburg Clinical Research Center for Specialized Types of Medical Care - Oncology | Moiseyenko, Vladimir Mikhaylovich | 3 | Clinical Research Center Of Specialized Types Medical Care-Oncology IRB/IEC |
| Argentina | Centro Oncologico Riojano Integral | Kaen, Diego Lucas | 3 | Comite Independiente de Etica para Ensayos en Farmacologia Clinica |
| Colombia | Administradora Country S.A. | Franco, Sandra | 3 | Comite de Etica en Investigacion Clinica - Clinica del Country |
| Australia | Macquarie University Hospital | Kefford,  Richard | 3 | Macquarie University Human Research Ethics Committee Medical Sciences |
| Malaysia | University Malaya Medical Centre | Bustam, Anita Zarina | 3 | Medical Ethics Committee (UMMC) University Malaya Medical Centre (UMMC) |
| Philippines | Tamayo Makati Medical Center | Belen, Maria | 3 | Makati Medical Center IRB/IEC |
| Taiwan | National Taiwan University Hospital | Lu, Yen-Shen | 3 | National Taiwan University Hospital - Research Ethics Committee IRB/IEC |
| Thailand | Ramathibodi Hospital | Ativitavas, Touch | 3 | Ethical Clearance Committee on Human Rights |
| Japan | National Hospital Organization Osaka National Hospital | Masuda, Norikazu | 3 | National Hospital Organization Osaka National Hospital IRB |
| Japan | Aichi Cancer Center Hospital | Iwata, Hiroji | 3 | Aichi Cancer Center Hospital Institutional Review Board |
| Japan | Kumamoto University Hospital | Iwase, Hirotaka/ Yamamoto, Yutaka | 3 | Kumamoto University Hospital Institutional Review Board |
| U.S.A. | Duke Breast Oncology Research | Blackwell, Kimberly/Force, Jeremy | 2 | Duke University Health System Institutional Review Board Office |
| U.S.A. | Sidney Kimmel Comprehensive Cancer Center at Johns Hopkins | Connolly, Roisin/ Wolff, Antonio C. | 2 | Johns Hopkins Medicine Institutional Review Boards |
| U.S.A. | University of Louisville, James Graham Brown Cancer Center | Riley, Elizabeth | 2 | University of Louisville – Institutional Review Board |
| U.S.A. | Texas Oncology | Patt, Debra A. | 2 | US Oncology, Inc. IRB |
| U.S.A. | Cancer Care Centers of South Texas | Wilks, Sharon T. | 2 | US Oncology, Inc. IRB |
| U.S.A. | Nebraska Cancer Specialists | Block, Margaret | 2 | US Oncology, Inc. IRB |
| Netherlands | Universitair Medisch Centrum Groningen | Schroder, C.P. | 2 | Stichting BEBO |
| Netherlands | Reinier de Graaf Gasthuis | Beelen, Karin | 2 | Stichting BEBO |
| Spain | Hospital Arnau de Vilanova | Llombart Cussac, Antonio | 2 | Hospital Vall D Hebron |
| Sweden | Onkologiska Kliniken | Linderholm, Barbro | 2 | Regionala etikprovningsnamnden |
| Sweden | Karolinska Universitetssjukhuset | Bjohle, Judith/ Foukakis, Theodoros | 2 | Regionala etikprovningsnamnden |
| Sweden | Skanes Universitetssjukhus | Killander, Fredrika | 2 | Regionala etikprovningsnamnden |
| Switzerland | Kantonsspital Winterthur | Mueller, Andreas | 2 | Kantonale Ethikkommission Zuerich - KEK |
| Hong Kong | Prince of Wales Hospital | Yeo, Winnie | 2 | Joint CUHK-NTEC Clinical Research  Ethics Committee |
| Russia | Scientific Research Oncology Institute n.a. N.N. Petrov | Krivorotko, Petr Vladimirovich | 2 | LEC of Scientific Research Oncology  Institute n.a. N.N.Petrov |
| Russia | Chelyabinsk Regional Clinical Oncological Dispensary | Fadeeva, Natalia Vladimirovna | 2 | Chelyabinsk Regional Clinical Oncological Dispensary IRB/IEC |
| Argentina | Instituto de Oncologia de Rosario | Fein, Luis | 2 | Comite de Etica Dr. Claude Bernard |
| Brazil | A.C. Camargo Cancer Center | Cordeiro de Lima, Vladmir | 2 | Comite de Etica em Pesquisa da Fundacao Antonio Prudente Hospital do Cancer A. C. Camargo  CONEP - Comissao Nacional de Etica  em Pesquisa |
| Guatemala | Grupo Medico Angeles | Castro, Hugo Raul | 2 | Zugueme |
| Peru | Instituto Oncologico Miraflores | Fuentes Rivera, Hugo Alejandro | 2 | Comite de Etica Asociación Benefica  Prisma |
| Peru | Instituto Nacional de Enfermedades Neoplásicas | Gomez, Henry | 2 | Comite Institucional de Etica en Investigacion del INEN |
| Australia | Monash Cancer Centre | White, Michelle | 2 | Monash Health Human Research Ethics Committee |
| New Zealand | Waikato Hospital - Oncology Clinical Trials | Kuper-Hommel, Marion | 2 | Southern Health and Disability Ethics  Committee |
| Malaysia | Hospital Kuala Lumpur | Mohamad Nor, Ibtisam | 2 | Medical Research & Ethics Committee (IMR) |
| Malaysia | Gleneagles Penang Clinical Research Center | Leong, Kin Wah | 2 | Medical Ethics & Research Committee (MREC) |
| Singapore | Johns Hopkins Singapore | Bharwani, Lavina | 2 | Singhealth Centralized Institutional Review Board |
| Japan | Social Medical Corporation Hakuaikai Sagara Hospital | Rai, Yoshiaki/ Sagara, Yasuaki | 2 | Social Medical Corporation Hakuaikai Sagara Hospital IRB |
| Japan | National Hospital Organization Shikoku Cancer Center | Hara, Fumikata/ Ohsumi, Shozo | 2 | National Hospital Organization Shikoku Cancer Center IRB |
| Japan | National Hospital Organization Kyushu Cancer Center | Tokunaga, Eriko | 2 | National Hospital Organization Kyushu Cancer Center IRB |
| U.S.A. | Cleveland Cancer Center | Baar, Joseph C. | 1 | University Hospital of Cleveland IRB |
| U.S.A. | Sanford Cancer Center Oncology Clinic | Dib, Elie/ Mazurczak,  Miroslaw A. | 1 | Western Institutional Review Board |
| U.S.A. | Northwest Cancer Specialists, P.C. | Smith, John W. | 1 | US Oncology, Inc. IRB |
| U.S.A. | Virginia Cancer Specialists | Irwin, Amy J. | 1 | US Oncology, Inc. IRB |
| U.S.A. | Texas Oncology, PA | Richards, Donald A. | 1 | US Oncology, Inc. IRB |
| U.S.A. | Oncology & Hematology Associates of Southwest Virginia, Inc., DBA Blue Ridge Cancer Care | Richards, Paul D. | 1 | US Oncology, Inc. IRB |
| Germany | Med. Centrum fuer Haematologie und Onkologie Bethanien | Loibl, Sibylle | 1 | Universitaet Erlangen Nuernberg |
| Netherlands | Medisch Centrum Haaglanden Westeinde | Oosterkamp, Rianne | 1 | Stichting BEBO |
| Netherlands | Vereniging Het Nederlands Kanker Instituut | Linn, Sabine | 1 | Stichting BEBO |
| Poland | Zachodniopomorskie Centrum Onkologii | Wysocki, Piotr Jan/Foszczynska-Kloda, Malgorzata | 1 | Centrum Onkologii-Instytut im. Marii  Skłodowskiej-Curie |
| Switzerland | Ospedale San Giovanni Via Ospedale, Istituto Oncologico della Svizzera Italiana Bellinzona | Pagani, Olivia | 1 | Comitato Etico Cantonale |
| Switzerland | Klinik Engeried | Buser, Katharina/ Borner, Markus M. | 1 | Comitato Etico Cantonale |
| South Africa | University of Pretoria Steve Biko Academic Hosp. | Dreosti, Lydia Mary | 1 | University of Pretoria – Research Ethics Committee |
| South Africa | Groote Schuur Hospital | Mohamed, Zainab | 1 | University of Cape Town Human Research Ethics Committee (HREC) |
| South Africa | Oncology Centre Vincent Pallotti Hospital | Hall, Jacqueline | 1 | Pharma Ethics |
| South Africa | Wits Clinical Research, Charlotte Maxeke Johannesburg Academic Hospital | Demetriou, Georgia Savva | 1 | University of Witwatersrand Human  Research Ethics Committee |
| Turkey | Acibadem Altunizade Hastanesi | Basaran, Gul | 1 | Ege Uni. Tip Fakultesi Arastirma Etik Kurulu |
| Argentina | Sanatorio Britanico | Tatangelo, Marcelo Daniel | 1 | Comite de Ética del Sanatorio Britanico S.A |
| Argentina | Centro de Oncologia e Investigacion Buenos Aires COIBA | Varela, Mirta | 1 | Comite de Etica COIBA |
| Brazil | Hospital Araujo Jorge Associacao de Combate ao Cancer de Goias | de Freitas, Ruffo, Jr. | 1 | Comite de Etica em Pesquisa da Associacao de Combate ao Cancer de  Goias – ACCG  CONEP - Comissao Nacional de Etica  em Pesquisa |
| Guatemala | Private Clinic Mario Fredy Sandoval | Sandoval, Mario Fredy | 1 | Zugueme |
| Guatemala | Oncomedica | Lopez, Karla Alejandra | 1 | Comite de Etica Independiente Zugueme |
| Guatemala | Centro Regional de Sub Especialidades Medicas | Ramirez, Julio Roberto | 1 | Zugueme |
| Australia | Chris O’Brien Lifehouse | McNeil, Catriona | 1 | Royal Prince Alfred Hospital IRB/IEC |
| Australia | Haematology & Oncology Clinics of Australia | McCarthy, Nicole | 1 | Bellberry Limited |
| Taiwan | China Medical University Hospital | Chiu, Chang-Fang | 1 | China Medical University Hospital Research Ethics Committee |
| Thailand | Chulalongkorn Hospital | Parinyanitikul, Napa | 1 | Institutional Review Board Faculty of Medicine Chulalongkorn University |
| Japan | Niigata Cancer Center Hospital | Sato, Nobuaki | 1 | Niigata Cancer Center Hospital Institutional Review Board |
| Japan | KKR Tohoku Kosai Hospital | Hirakawa, Hisashi | 1 | KKR Tohoku Kosai Hospital Institutional Review Board |
| U.S.A. | Kaiser Permanente Southern California | Polikoff, Jonathan | 1 | Kaiser Permanente Southern California |
| U.S.A. | West Virginia University | Mehmi, Inderjit/ Kurian, Sobha | 1 | West Virginia University IRB |
| U.S.A. | Fort Wayne Medical Oncology and Hematology | Chitneni,  Shalini | 1 | Western Institutional Review Board |
| U.S.A. | Memorial Sloan Kettering Cancer Center | Gucalp, Ayca | 1 | Memorial Sloan Kettering Cancer Center IRB |

|  | **Section/topic** | **No** | **CONSORT 2025 checklist item description** | **Reported on page no.** |
| --- | --- | --- | --- | --- |
|  | **Title and abstract** | | |  |
|  | Title and structured abstract | 1a | Identification as a randomised trial | 4 |
|  |  | 1b | Structured summary of the trial design, methods, results, and conclusions | - |
|  | **Open science** | | |  |
|  | Trial registration | 2 | Name of trial registry, identifying number (with URL) and date of registration | 4, 17 |
|  | Protocol and statistical analysis plan | 3 | Where the trial protocol and statistical analysis plan can be accessed | - |
|  | Data sharing | 4 | Where and how the individual de-identified participant data (including data dictionary), statistical code and any other materials can be accessed | 21 |
|  | Funding and conflicts of interest | 5a | Sources of funding and other support (eg, supply of drugs), and role of funders in the design, conduct, analysis and reporting of the trial | 22 |
|  |  | 5b | Financial and other conflicts of interest of the manuscript authors | 22-26 |
|  | **Introduction** | | |  |
|  | Background and rationale | 6 | Scientific background and rationale | 5-6 |
|  | Objectives | 7 | Specific objectives related to benefits and harms | 6 |
|  | **Methods** | | |  |
|  | Patient and public involvement | 8 | Details of patient or public involvement in the design, conduct and reporting of the trial | - |
|  | Trial design | 9 | Description of trial design including type of trial (eg, parallel group, crossover), allocation ratio, and framework (eg, superiority, equivalence, non-inferiority, exploratory) | 18 |
|  | Changes to trial protocol | 10 | Important changes to the trial after it commenced including any outcomes or analyses that were not prespecified, with reason | - |
|  | Trial setting | 11 | Settings (eg, community, hospital) and locations (eg, countries, sites) where the trial was conducted | 7 |
|  | Eligibility criteria | 12a | Eligibility criteria for participants | 17 |
|  |  | 12b | If applicable, eligibility criteria for sites and for individuals delivering the interventions (eg, surgeons, physiotherapists) | - |
|  | Intervention and comparator | 13 | Intervention and comparator with sufficient details to allow replication. If relevant, where additional materials describing the intervention and comparator (eg, intervention manual) can be accessed | 18 |
|  | Outcomes | 14 | Prespecified primary and secondary outcomes, including the specific measurement variable (eg, systolic blood pressure), analysis metric (eg, change from baseline, final value, time to event), method of aggregation (eg, median, proportion), and time point for each outcome | 19 |
|  | Harms | 15 | How harms were defined and assessed (eg, systematically, non-systematically) | - |
|  | Sample size | 16a | How sample size was determined, including all assumptions supporting the sample size calculation | - |
|  |  | 16b | Explanation of any interim analyses and stopping guidelines | - |
|  | Randomisation: |  |  |  |
|  | Sequence generation | 17a | Who generated the random allocation sequence and the method used | - |
|  |  | 17b | Type of randomisation and details of any restriction (eg, stratification, blocking and block size) | 18 |
|  |  |  |  |  |
|  |  |  |  | **Reported on page no.** |
|  | Allocation concealment mechanism | 18 | Mechanism used to implement the random allocation sequence (eg, central computer/telephone; sequentially numbered, opaque, sealed containers), describing any steps to conceal the sequence until interventions were assigned | - |
|  | Implementation | 19 | Whether the personnel who enrolled and those who assigned participants to the interventions had access to the random allocation sequence | - |
|  | Blinding | 20a | Who was blinded after assignment to interventions (eg, participants, care providers, outcome assessors, data analysts) | - |
|  |  | 20b | If blinded, how blinding was achieved and description of the similarity of interventions | - |
|  | Statistical methods | 21a | Statistical methods used to compare groups for primary and secondary outcomes, including harms | - |
|  |  | 21b | Definition of who is included in each analysis (eg, all randomised participants), and in which group | 19 |
|  |  | 21c | How missing data were handled in the analysis | - |
|  |  | 21d | Methods for any additional analyses (eg, subgroup and sensitivity analyses), distinguishing prespecified from post hoc | 18-20 |
|  | **Results** | | |  |
|  | Participant flow, including flow diagram | 22a | For each group, the numbers of participants who were randomly assigned, received intended intervention, and were analysed for the primary outcome | 7 |
|  |  | 22b | For each group, losses and exclusions after randomisation, together with reasons | - |
|  | Recruitment | 23a | Dates defining the periods of recruitment and follow-up for outcomes of benefits and harms | 7 |
|  |  | 23b | If relevant, why the trial ended or was stopped | - |
|  | Intervention and comparator delivery | 24a | Intervention and comparator as they were actually administered (eg, where appropriate, who delivered the intervention/comparator, how participants adhered, whether they were delivered as intended (fidelity)) | - |
|  |  | 24b | Concomitant care received during the trial for each group | - |
|  | Baseline data | 25 | A table showing baseline demographic and clinical characteristics for each group | - |
|  | Numbers analysed,  outcomes and estimation | 26 | For each primary and secondary outcome, by group:  ● the number of participants included in the analysis  ● the number of participants with available data at the outcome time point  ● result for each group, and the estimated effect size and its precision (such as 95% confidence interval)  ● for binary outcomes, presentation of both absolute and relative effect size | - |
|  | Harms | 27 | All harms or unintended events in each group | - |
|  | Ancillary analyses | 28 | Any other analyses performed, including subgroup and sensitivity analyses, distinguishing pre-specified from post hoc | 7-11 |
|  | **Discussion** | | |  |
|  | Interpretation | 29 | Interpretation consistent with results, balancing benefits and harms, and considering other relevant evidence | 11-16 |
|  | Limitations | 30 | Trial limitations, addressing sources of potential bias, imprecision, generalisability, and, if relevant, multiplicity of analyses | - |

Citation: Hopewell S, Chan AW, Collins GS, Hróbjartsson A, Moher D, Schulz KF, et al. CONSORT 2025 Statement: updated guideline for reporting randomised trials. BMJ. 2025; 388:e081123. <https://dx.doi.org/10.1136/bmj-2024-081123>
© 2025 Hopewell et al. This is an Open Access article distributed under the terms of the Creative Commons Attribution License (<https://creativecommons.org/licenses/by/4.0/>), which permits unrestricted use, distribution, and reproduction in any medium, provided the original work is properly cited.

*We strongly recommend reading this statement in conjunction with the CONSORT 2025 Explanation and Elaboration and/or the CONSORT 2025 Expanded Checklist for important clarifications on all the items. We also recommend reading relevant CONSORT extensions. See [www.consort-spirit.org](http://www.consort-spirit.org).
